# Retraction: Congenital Hydronephrosis Due to Pyeloureteral Atresia: A Case Report

**DOI:** 10.7759/cureus.r182

**Published:** 2025-05-29

**Authors:** Zainab H Alowainati, Zainab H Salman, Hibah A Alhamad, Njood Alsudairy

**Affiliations:** 1 College of Medicine, Jordan University of Science and Technology, Irbid, JOR; 2 College of Medicine, Cairo University, Cairo, EGY; 3 College of Medicine, Imam Abdulrahman Bin Faisal University, Dammam, SAU; 4 Radiology, The Second Health Cluster, Jeddah, SAU

This article has been retracted by the Editors-in-Chief due to a significant discrepancy identified after publication. After the initial submission and peer review process, the authors replaced the article’s content with unrelated material discussing intussusception, which does not align with the original title and topic of congenital hydronephrosis. The journal editorial office greatly regrets that this was not identified prior to publication.

